# Meditation and Metacontrol

**DOI:** 10.1007/s41465-017-0017-4

**Published:** 2017-04-04

**Authors:** Bernhard Hommel, Lorenza S. Colzato

**Affiliations:** 1 Cognitive Psychology Unit & Leiden Institute for Brain and Cognition, Leiden University, Wassenaarseweg 52, 2333 AK Leiden, The Netherlands; 2 Department of Cognitive Psychology, Institute of Cognitive Neuroscience, Faculty of Psychology, Ruhr University Bochum, Bochum, Germany; 3 Institute for Sports and Sport Science, University of Kassel, Kassel, Germany

**Keywords:** Cognitive control, Focused-attention meditation, Open-monitoring meditation, Decision making, Persistence, Flexibility

## Abstract

In addition to longer-term engagement in meditation, the past years have seen an increasing interest in the impact of single bouts of meditation on cognition. In this hypothesis and theory article, we adopt the distinction between focused-attention meditation (FAM) and open-monitoring meditation (OMM) and argue that these different types of meditation have different, to some degree, opposite impact on cognitive processes. We discuss evidence suggesting that single bouts of FAM and OMM are sufficient to bias cognitive control styles towards more versus less top-down control, respectively. We conclude that all meditation techniques are not equal and that successful meditation-based intervention requires the theoretically guided selection of the best-suited technique.

## Introduction

While meditation is often viewed and employed as a technique to reduce stress (Chiesa and Seretti 2009), its potential to increase cognitive abilities has been emphasized from its beginnings (e.g., Luk 1994). For instance, some Buddhist meditation techniques explicitly and intentionally target the training and improvement of concentration (manasikara) and insight (vipassanā; see Santucci 1979), which tap into attentional control and higher-order cognition that according to Lutz et al. (2008) are systematically affected and improved by meditation. These possible links to cognitive improvement have attracted the attention of researchers, who indeed found evidence that active meditators show improved performance in a whole range of cognitive tasks. In particular, engaging in focused-attention meditation (FAM; which typically calls for sustaining selective attention moment-by-moment on a specific object with a fairly narrow focus: Lutz et al. 2015), open-monitoring meditation (OMM; which typically calls for the attentive monitoring of anything that occurs in experience without focusing on any explicit object), and loving-kindness or compassion meditation has been found to be associated with specific effects on attentional selection, conflict monitoring, and creativity-related tasks (Lippelt et al. 2014). However, active meditators can be assumed to have a positive bias regarding the usefulness of meditation and they are unlikely to be representative of the general population regarding their personality and affective attitude towards meditation. Overcoming such methodological difficulties requires extended training studies with appropriate control groups and random assignment of participants to meditation and control training (Green et al. 2014), which renders research demanding and expensive.

Interestingly, however, recent studies suggest that meditation can impact cognitive abilities without extended practice. Just like for long-term effects, commonly from studies spanning weeks or months, or more (Lippelt et al. 2014), these short-term effects (from studies spanning no more than 1–2 h) have been reported for attentional selection, conflict monitoring, and creativity-related tasks, and FAM and OMM were found to have opposite effects. With regard to attentional selection, a single bout of FAM was shown to induce a larger attentional blink than a single bout of OMM (Colzato et al. 2015a). Given that this effect is likely to reflect over-selectivity (i.e., the too-exclusive allocation of attentional resources to the first of two to-be-reported targets: Olivers and Nieuwenhuis 2005, 2006), this observation suggests that FAM is more effective in promoting the focusing on a single target than OMM is.

With respect to conflict monitoring, engaging in OMM reduced the degree to which participants engaged in trial-to-trial control adjustments, as compared to performance after engaging in FAM (Colzato et al. 2015b). Control adjustments are assumed to be reflected by the observation that effects of stimulus-response compatibility (i.e., leakages of attention that lead to response conflict) are reduced or even absent after incompatible trials (Gratton et al. 1992). This reduction has been attributed to increased focusing on the task goal (Botvinick et al. 2001), which implies that focusing on this goal is more effectively promoted by FAM than it is by OMM.

As for creativity, OMM, but not FAM, improves brainstorming-like divergent thinking as assessed by the Alternate Uses Task (Guilford 1967) in both experienced meditation practitioners (Colzato et al. 2012b) and novices (Colzato et al. 2017). FAM, but not OMM, tended to improve performance in persistence-heavier convergent-thinking tasks like the Remote Association Task (Mednick and Mednick 1967), but these effects were weak and not replicable (Colzato et al. 2017). The most likely reason for that is that participants were mainly university students, who arguably engage in convergent thinking all day (at least when visiting the university), so that interventions to improve that ability further are likely to be futile.

There is thus evidence that even briefly engaging in meditative activity is sufficient to improve or impair performance in a number of cognitive tasks, even in individuals that have no previous experience with meditation. Given the brevity of the meditational experience, we assume that these effects do not reflect the acquisition of a particular skill but, rather, the establishment of a particular cognitive state. In the following, we indeed suggest that, possible long-term effects notwithstanding, engaging in FAM and OMM induces particular metacontrol states, that is, states that moderate the way that individuals exert cognitive control on lower-level processes. To motivate this suggestion, we will first introduce the concept of a metacontrol state and then apply this concept to meditation.

## Cognitive Control

**Fig. 1:**
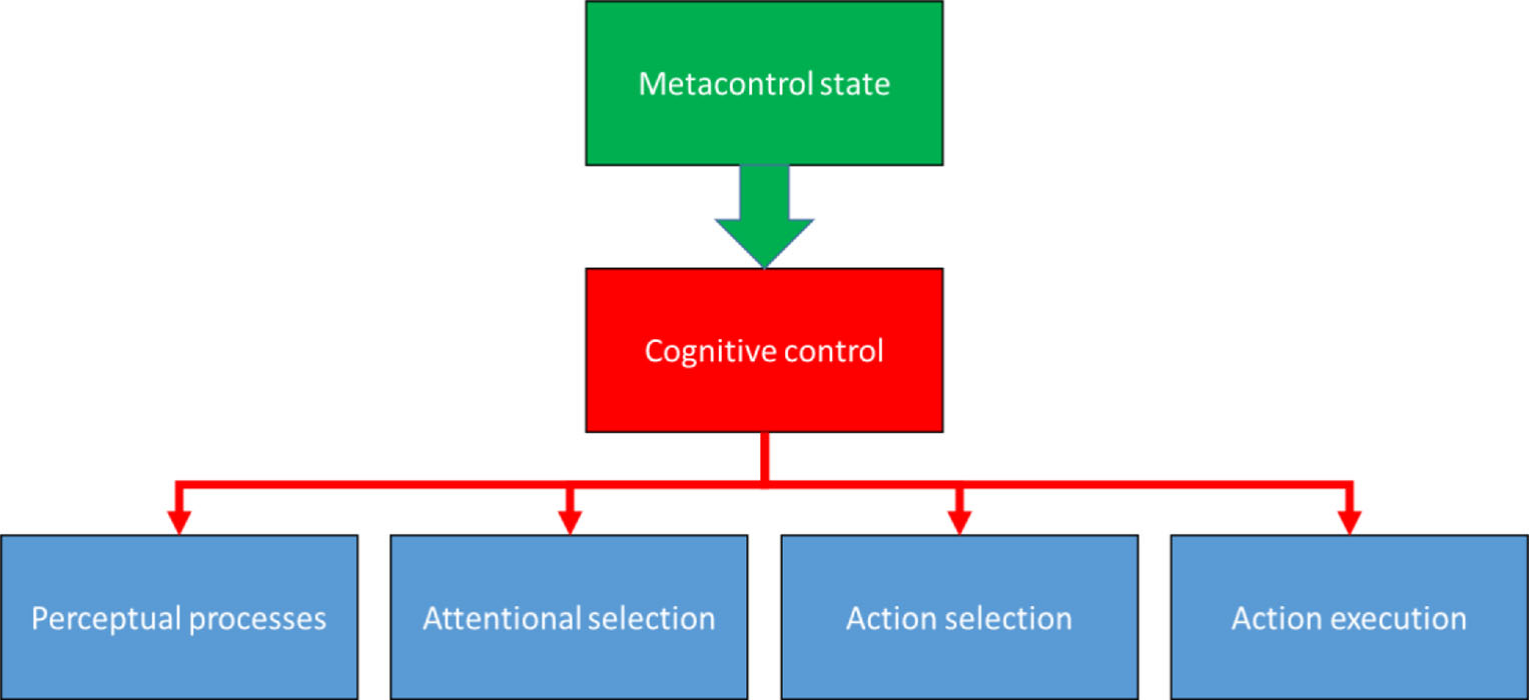
ᅟConcept of a metacontrol state

Modern functional theories of human cognition and behavior commonly attribute psychological phenomena to the characteristics of human information processing (Neisser 1967). Stimulus information provided by the environment is assumed to undergo perceptual analysis and attentional selection, which might be followed by selecting and executing a particular action—as sketched in lowest level of Fig. 1. Various weaknesses and shortcomings of the information-processing metaphor (cf., Hommel et al. 2001) notwithstanding, functional theorists agree on the assumption that stimulus input undergoes various processes before overt actions are issued. However, they also agree on the insight that effective, contextually adjusted performance requires the configuration of the functional systems performing these cognitive processes to the task at hand—an ability often referred to as cognitive control or executive function (see the second, middle layer of Fig. 1).

The concept of cognitive control can be seen as a more mechanistic version of the outdated concept of will (Goschke 2003), and it is fair to say that we currently know considerably more about *what* cognitive control is doing than about *how* it actually works (e.g., Monsell 2003). Interestingly for our purposes, however, there is increasing evidence that control may not be exerted by a dedicated unitary system or brain area, but that it rather emerges through the interplay of multiple systems and areas. Of particular importance are contributions of two dopaminergic pathways: the mesofrontal pathway originating in the ventral tegmental area and the nigrostriatal pathway originating in the substantia nigra (Cools 2006). These two pathways are assumed to underlie different and, to some degree, opposing cognitive control functions: while the mesofrontal pathway seems to be important for working memory and the representation and maintenance of goals, the nigrostriatal pathway seems to play a key role in inhibiting ongoing actions and taking in external information (Cools 2006, 2008).

Goschke (2000) has suggested that the interplay if not struggle between control systems with such opposing functionalities might reflect and provide a solution for what he calls the stability-flexibility dilemma that humans are facing. On the one hand, effectively performing complex, temporally extended actions requires some degree of stability of the underlying goal and task set, which in turn calls for a considerable persistence of cognitive control. On the other hand, however, humans are living in a rapidly changing environment, so that a planned or ongoing action may quickly turn out to be no longer appropriate or unlikely to overcome unforeseen obstacles. Too much persistence would thus not be adaptive. Cognitive control would thus benefit from a system that keeps monitoring environmental conditions to check whether the requirements of the ongoing action are still given and whether interesting alternatives are available. In case that the originally intended action is no longer useful or less appropriate than other available alternatives, this system could then stall the ongoing action and facilitate the implementation of another action goal. Hence, context-sensitive cognitive control may emerge from the interaction between (systems promoting) persistence and flexibility.

## Metacontrol

The existence of multiple interacting systems that create cognitive control as an emergent property raises the question of how this interaction is controlled. The probably most intuitive move would be to reinterpret this interaction in terms of the well-established dual-route approach to decision-making and action control, according to which choices emerge from the struggle between will and habit or, in more modern terms, between intentional and automatic routes (Bargh 1989). This would suggest that the mesocortical persistence system would rely on and represent the intention of the agent while the nigrostriatal flexibility system would be driven by and represent stimulus information. If so, only one of the two routes or systems would actually underlie cognitive control proper.

**Fig. 2:**
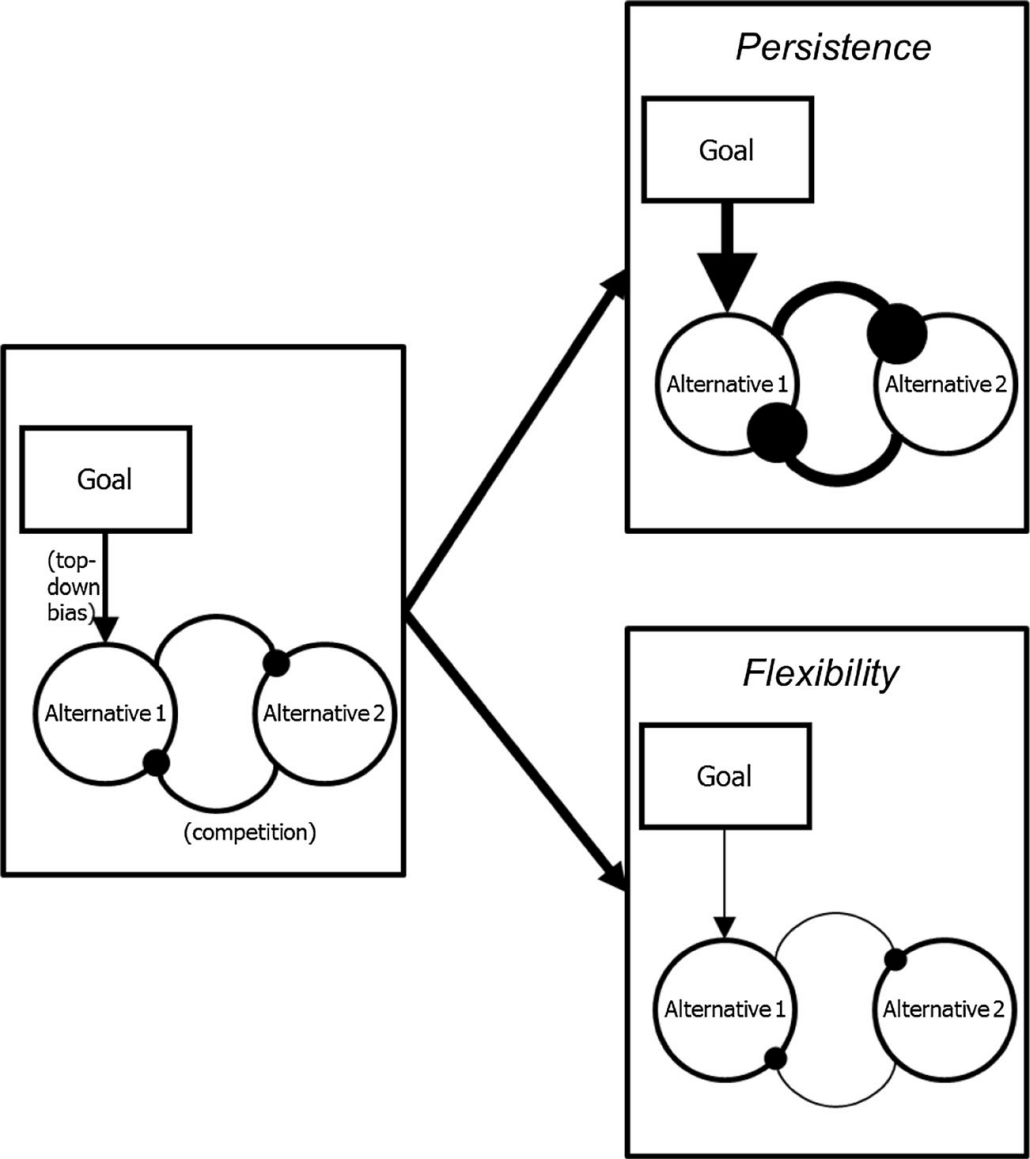
ᅟCore ingredients of biologically plausible decision-making models

Converging evidence suggests another possible scenario however. As discussed in more detail elsewhere (Hommel 2000, 2015), humans have control over the degree to which the “intentional” and the “automatic” route have impact on their decisions. For instance, the Simon effect (better performance if spatial responses correspond to task-irrelevant location of a stimulus) is commonly assumed to reflect automatic processing (for a review, see Hommel 2011), and it has in fact been shown that presenting a lateralized stimulus creates a lateralized readiness potential in the opposite hemisphere (Eimer 1998). Interestingly, however, these signs of automaticity disappear if the stimulus is presented before the relevant stimulus-response mapping (Valle-Inclán and Redondo 1998), suggesting that automaticity is contingent on the task goal (Hommel 2010). Likewise, the size of the Simon effect is drastically reduced, if not eliminated after stimulus-response noncorresponding trials (Stürmer et al. 2002), suggesting that automaticity is fully controlled (a *prepared reflex* in the sense of Hommel 2000)—which obviously renders the term nonsensical. Couching these observations in terms of persistence and flexibility provides a more coherent picture. Let us consider how a bias towards persistence or flexibility might affect human decision-making. Figure 2 shows the core ingredients of biologically plausible decision-making models (for a review and critical evaluation, see Bogacz 2007). The leftmost panel shows representations of two alternatives (1 and 2) competing for a decision, as well as the two basic principles that characterize human decision-making. First, it is competitive, in the sense that the relative strength or activation of one alternative determines its competitiveness and the degree to which it suppresses (and eventually outcompetes) other alternatives—this is indicated by the two mutually inhibitory links between the two alternatives. Second, decision-making is biased by goal representations, in the sense that more goal-compatible alternatives receive more support and are thus more likely to outcompete less goal-compatible alternatives—this is indicated by the facilitatory arrow from the goal representation that in the example supports alternative 1.

Let us now consider how a stronger bias towards persistence would affect this scenario. As indicated in the top-right panel, more persistence would be achieved by increasing the top-down bias from the goal representation and/or the degree of competition between alternatives (two possibilities that have not yet been disentangled empirically; see Hommel 2015). Decision-making would become more selective, and irrelevant (i.e., not goal-compatible) information would be more effectively excluded. It is easy to see that this would, among other things, reduce or eliminate the Simon effect, suggesting that previous demonstrations that it can indeed be eliminated are likely to reflect a strong bias towards persistence. A strong bias towards flexibility would be achieved by the exact opposite, namely, by reducing the top-down bias and the degree of competition (see bottom-right panel), which in the most extreme case would lead to indecision. It is easy to see that this would lead to a much more “democratic” decision mode that provides more opportunities for irrelevant information.

From a persistence-stability point of view, it is tempting to replace the semantically and empirically unconvincing idea that only one of the two players in decision-making is actually controlled with a scenario according to which people actually control both players by creating a particular balance between persistence and flexibility. The control over this balance can be considered the control of cognitive control, which is why Hommel (2015) refers to it as *metacontrol* and the current degree of persistence or flexibility as *metacontrol state*. Note that this concept is agnostic with respect to the degree of consciousness, awareness, or phenomenal experience that may or may not go with particular metacontrol states (issues emphasized by meta-awareness approaches; e.g., Chin and Schooler 2010; Lutz et al. 2015) but only considers the functional and neural characteristics of metacontrol states. Interestingly, there is evidence that metacontrol states can become chronified, resulting in default biases towards persistence or flexibility, as a function of the individual genetic profile (Markett et al. 2011) and cultural norms and values (Masuda and Nisbett 2001). Even more important for our purposes, there is also evidence for short-term priming, in the sense that the current metacontrol state of individuals can systematically be biased by experimental manipulations.

For instance, positive affect has been suspected to induce a bias towards flexibility (Dreisbach and Goschke 2004), which fits with the observation that inducing positive affect increases the spontaneous eyeblink rate, a clinical indicator of the individual dopamine level in the nigrostriatal pathway (Akbari Chermahini and Hommel 2012). If so, one would expect that inducing positive mood improves performance in tasks that rely on flexibility but impairs performance in tasks that rely on persistence. Indeed, positive-mood induction increases brainstorming performance as assessed by the Alternate Uses Task and related creativity tests (Baas et al. 2008), but it also leads to less systematic attentional focusing and greater distractibility in selective attention tasks (Dreisbach and Goschke 2004). Inducing positive mood and presenting unexpected reward have also been shown to reduce or even eliminate trial-to-trial adjustments of top-down control (van Steenbergen et al. 2010), apparently by attenuating the response of the anterior cingulate cortex (a system that is assumed to upregulate top-down control in the presence of conflict: Botvinick et al. 2001) to stimulus-induced conflict (Botvinick et al. 2001). Negative mood, in turn, has been reported to increase top-down control, as in states of dysphoria induced by acute tryptophan depletion in remitted depressed patients (Booij et al. 2005). Along the same lines, inducing negative mood increases the shielding of a prioritized task against competing tasks, as indicated by a reduction of cross talk between tasks (Zwosta et al. 2013). Taken altogether, there is systematic evidence that positive-going mood drives metacontrol towards flexibility and some evidence that negative-going mood promotes persistence.

Given that the subjective experience of mood correlates with, and presumably relies on the dopamine level of the nigrostriatal pathway (Ashby et al. 1999), one may wonder whether it is the conscious experience or, as our approach would suggest, the underlying neurotransmitter activity that is responsible for metacontrol biases. Preliminary evidence suggests the latter: Akbari Chermahini and Hommel (2012) reported that the mere performance of a task requiring persistence or flexibility is sufficient to induce a corresponding mood change; i.e., mood is improved when engaging in divergent thinking but worsened when engaging in convergent thinking. In other words, the mood experience does not seem to be the cause but the result (or the conscious reflection) of dopamine-regulated metacontrol biases.

Other manipulations were also successful in inducing particular metacontrol biases. For instance, interleaving a dual task with a persistence-heavy convergent-thinking task has been found to reduce inter-task cross talk in the former (Fischer and Hommel 2012). Social attention and attitudes also seem to be sensitive to manipulations targeting metacontrol. For instance, interleaving a joint Simon task with a flexibility-heavy divergent-thinking task increases the degree to which the actions of a co-agent are considered when deciding about one’s own actions (Colzato et al. 2013), and the same effect is obtained when the joint Simon task is carried out after having encircled all relational pronouns (as compared to all personal pronouns) in a text (Colzato et al. 2012a). Performing in a divergent-thinking task was also found to increase interpersonal trust (Sellaro et al. 2014). Taken altogether, there is converging evidence that various factors that suggest, promote, or require more persistence or more flexibility have a systematic effect on the way that other tasks are performed, in the sense that biasing the system towards persistence improves performance in persistence-heavy tasks but impairs performance in flexibility-heavy tasks, while biasing the system towards flexibility has the opposite effect. We take that to imply that metacontrol states exist (as they seem to affect other tasks than the one they were implemented for) and that they are flexible to at least some degree—presumably within constraints determined by the genetic setup and the cultural background (Hommel 2015).

## Meditation and Metacontrol

Now that we have introduced the concept of metacontrol states, let us return to the question of how engaging in meditation practices affects human cognition and performance. We suggest that, first, engaging in meditation induces an immediate bias of the metacontrol state and that, second, different meditation practices can induce biases that differ in direction. Take FAM, which has the purpose to have the meditator focus on one single item, one mental content at a time, and which uses instructions serving that purpose. It makes sense to assume that these instructions are effective in biasing the metacontrol state of the meditator towards persistence which, depending on the strength of the bias, is likely to improve the meditator’s performance in tasks that require persistence but not in tasks that require flexibility. Reversely, OMM has the purpose to have the meditator process multiple items, multiple mental contents in parallel, or at least close in time and thus uses instructions achieving that. It makes sense to assume that these instructions effectively bias the metacontrol state towards flexibility, so that performance in flexibility-heavy tasks, but not in persistence-heavy tasks, should benefit.

This emerging scenario fits well with the available findings. For the attentional blink, it would predict a more pronounced blink related to FAM than to OMM, or vice versa: FAM-induced stronger persistence should further increase the tendency to allocate attention to the first target, so that the second is even more likely to be ignored, while OMM-induced flexibility should have the opposite effect. This is indeed what has been reported from both long-term and short-term (see Lippelt et al. 2014, for a review) studies. For conflict monitoring, one would expect that engaging in FAM leads to more monitoring and top-down control adjustments than engaging in OMM, which again fits with findings from both long-term (Tang et al. 2007) and short-term (Colzato et al. 2015b) studies. Finally, FAM would go better with top-down guided convergent thinking while OMM should be more compatible with associative brainstorming, which again fits with both long-term (Lebuda et al. 2016) and short-term (Colzato et al. 2012b, 2017) studies.

Taken altogether, we consider our metacontrol hypothesis a useful tool to make sense of both short-term and long-term effects of meditation and to generate directed predictions for FAM-type and OMM-type meditation techniques. This is not to say that our account represents an exhaustive theory of meditation already, as a number of questions remain.

## Open Questions

An important question of both theoretical and practical relevance is how our considerations translate into existing meditation techniques. FAM and OMM can be considered pure cases that in practice are often mixed and often intentionally so—just think of Mindfulness-Based Stress Reduction (MBSR), Mindfulness-Based Cognitive Therapy (MBCT), and Insight (aka Vipassana) meditation. Indeed, it is likely that focusing requires a mind state that is more familiar to Western individuals than open monitoring, and it may be easier to understand how to “let go” top-down control (as required for OMM) after having engaged in practicing FAM. This raises the question of how to predict behavior for these mixed-type interventions. Our approach suggests that it is the current state that matters, irrespective of previous experience and previously adopted mind state. But it is also conceivable that repeatedly engaging in and switching between mind states that differ in terms of persistence and flexibility promote some sort of higher-order flexibility by allowing the individual to rapidly adopt the mind state that is most suitable for a given situation. Looking into pure cases only, as in the studies that we have discussed here, might lead one to overlook this possibility.

For instance, in most studies that compared FAM and OMM, one of the two techniques had a stronger effect than the other, often with the other showing weak or no significant effects. While some of these asymmetries might be due to existing metacontrol biases in the investigated population (e.g., university students), it would be more convincing if such preexperimental biases could be objectively assessed and predicted. Furthermore, while the observed effects fit with our suggested scenario, one might wonder why other, apparently similar effects have not been found. For instance, Colzato et al. (2015b) observed an effect of meditation on conflict monitoring in a Simon task, but the actual Simon effect was not affected. Should an increase in persistence (or flexibility) not reduce (increase) the processing of irrelevant information and, thus, reduce (increase) the Simon effect? One might argue that the Simon effect is too automatic to be prevented online or too strong, or the impact of meditation might be too mild to be visible in the data, but a better mechanistic understanding of the underlying operations seems necessary to back up such speculations. It is for these reasons that we believe that our metacontrol hypothesis is a step into the right direction, but that other steps need to follow.
